# Magnitude and predictors of undernutrition among children aged six to fifty nine months in Ethiopia: a cross sectional study

**DOI:** 10.1186/s13690-017-0198-4

**Published:** 2017-07-10

**Authors:** Hiwot Darsene, Ayele Geleto, Abebaw Gebeyehu, Solomon Meseret

**Affiliations:** 1 MCHN Directorate, Federal Ministry of Health, Addis Ababa, Ethiopia; 2 School of Public Health, College of Health and Medical Science, Haramaya University, Harar, Ethiopia; 3 Institute of Public Health, Gondar College of Health and Medical Science, University of Gonder, Gonder, Ethiopia

**Keywords:** Children, Stunting, Wasting, Underweight and undernutrition

## Abstract

**Background:**

Undernutrition among children continues to be a major public health problem in developing countries. In Ethiopian, 44% of under-five children were stunted while 29% and 10% were underweight and wasted respectively. However, predictors of undernutrition among children were not clearly known in the study area. Therefore, this study was aimed at determining prevalence and predictors of undernutrition among children aged 6–59 months in Hawassa town.

**Method:**

A community based cross-sectional study was conducted among 811 randomly selected children paired with their mothers/caregivers. Mothers/caregivers were interviewed to obtain social-demographic data and feeding practice. Anthropometric measurement was conducted to obtain anthropometric data. Data were entered into EPI info 6.04 and exported to SPSS 16 for analysis. Bivariate logistic regression analysis with Crude Odds Ratio at 95%CI was used to assess presence of association among variables. Multivariate logistic regression analysis with Adjusted Odds Ratio at 95%CI was conducted to determine predictors of undernutrition and association was declared significant at *p* ≤ 0.05.

**Result:**

The result of our study indicated that 39.3%, 15.8% and 6.3% of children were stunted, underweighted and wasted respectively. Multivariate logistic regression analysis identified male sex, mother older than 35 years, not fed on colostrum, cessation of breastfeeding before two years of age, frequency of complementary feeding per day and diarrheal morbidity in the last 12 months were statistically associated with stunting. Maternal education, family sizes and diarrheal morbidity in the past 12 months were significantly associated with underweight. Similarly, frequency of complementary feeding per day, age at cessation of breastfeeding, preceding birth interval and not fed on colostrum were associated to wasting.

**Conclusion:**

The prevalence of undernutrition; stunting, underweight and wasting, among under-five children is very common in the study area. Inappropriate feeding practice and diarrheal morbidity were found to be the main risk factors for undernutrition. Appropriate factor specific interventions including counseling on optimal child feeding practice and diarrhea prevention should be strengthened in the study area.

## Operational definition

Kebele: the smallest administrative structure with household number of nearly 500.

Sidaminga: the widely spoken language (local language) of the study area.

Chembelala: A well known traditional festival that is celebrated in the study area and considered as a new year.

Diarrheal morbidity: Passage of three or more loose or liquid stools per day (or more frequent passage than is normal for the individual).

## Background

Adequate nutrition is essential during childhood to ensure healthy growth, proper organ development and function, a strong immune system, and neurological and cognitive development. Children undernutrition continues to be a major public health problem in developing countries. Global data indicated that 60 million children are moderately malnourished while approximately 13 million children faced severe acute malnutrition. Globally, undernutrition contributes for more than one third of child deaths which can be prevented through public health interventions [1–3].

Undernutrition can affect children’s health and learning ability during their adulthood life. Children with undernutrition are usually suffered from chronic illnesses [4]. Survivors of malnutrition can suffer from impaired physical development and intellectual abilities, which in turn may diminish their working capacity with negative effects on economic growth. Child malnutrition may also lead to higher levels of chronic illness in adult life and these may have intergenerational effects, as malnourished females are more likely to give birth to low-weight babies [5].

Globally it is estimated that 35% of deaths among under-five children is attributed to under nutrition. Over two-thirds of these deaths, which are often associated with inappropriate feeding practices, occur during the first year of life. In developing countries nearly one-third of children are underweight or stunted. Under nutrition is a risk factor for infectious disease and deaths [6].

Ethiopia is a country with remarkable progress in reducing under-five mortality [5]. On the other hand, currently undernutrition among children is a common health problem in the country [7]. In Ethiopia, undernutrition is a major public health problem that occurs throughout full year round because of long term household food insecurity. Undernutrition is the underlying cause for 57% of child deaths in the country. Different studies conducted in Ethiopia, including the national data, indicated high prevalence of undernutrition among children. The Data from Ethiopian Demographic and Health Survey (EDHS) 2011 indicated that 44% of under-five children were stunted while 29% and 10% were underweight and wasted respectively [5].

Study done in Vietnam revealed that the prevalence of underweight, stunting and wasting was found to be 31.8%, 44.3% and 11.9%, respectively [8]. Similarly, 48% of under-five children in East Africa are stunted [9]. Many nutritional studies have demonstrated that in Ethiopia undernutrition is a serious problem with regional variations of 51.4% in Tigray, 41.4% in Oromia and 44.1 in Southern Nation, Nationality and People Region (SNNPR) [5].

Undernutrition in developing countries in general and in Ethiopia in particular is highly prevalent. Knowledge on the level of undernutrition and its contributing factors is therefore an important prerequisite for developing strategies of nutritional intervention. However, there is no recent study which identified prevalence and predictors of undernutrition in the study area. Therefore this study was designed to identify prevalence of undernutrition and associated factors among children aged 6–59 months in Hawassa town.

## Methods

### Study design and setting

A community based cross-sectional study was conducted among children aged six to fifty nine months paired with their mothers/caregivers in Hawassa town. Hawassa town is located at 275 km south of Addis Ababa, the capital of Ethiopia. It is the regional capital of SNNPRs with multi diverse ethnicity, language, culture and life style. The city administration has eight sub-cities and thirty two kebeles. Tulla sub-city is among the eight sub-cities found in Hawassa Town. Tulla sub-city has 12 kebeles. According to the 2007 national census it has a total population of 100,790 of which under- five children and children in the age group of 6–59 months constitute 15,723 and 15,219 respectively. This study was conducted from August to September 2012.

### Study participants

The study population of this study was the 811 randomly selected six to fifty nine months old children paired with their mothers/caregivers who lived at least for six months in Tulla sub-city, Hawassa town with exclusion of children with disabilities that poses difficulty during anthropometric measurements.

### Sample size and sampling technique

The sample size was calculated using a single population proportion formula [*n* = (Z α/2)^2^ p (1-p) / d2] [2]. In Ethiopia, 44.1% of under-five children are stunted (P) [5];95% confidence interval and 5% marginal of error (d) were used to calculate sample size. Since a multistage sampling technique was employed to identify study participants, a design effect of 2 was used [2] and 10% non-response (refusal and closed houses after three repeated visit) was considered. Accordingly, the final sample size was calculated to be *n* = [(1.96)^2^ (0.441x0.559]/(0.05)^2^ = 834.

**Fig. 1:**
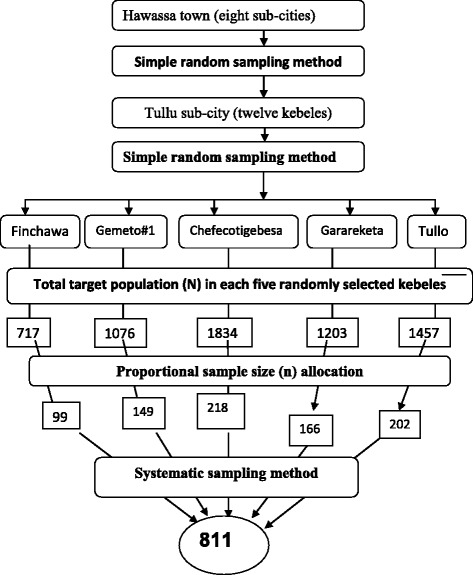
Schematic presentation of sampling technique, Hawassa town, Southern Ethiopia, 2012

A multistage sampling technique was used to identify study participants. First of all, Tullu sub-city was selected by simple random sampling (lottery) method from all sub-cities of Hawassa town. Then five Kebeles of Tulla sub-city (Tullo, Finchawa, Gemeto, Chefecotigebesa and Garareketa) were selected from the twelve kebeles of the sub-city again by lottery method. An estimated 5,842 households with eligible children were found in the selected kebeles. The sample size was proportionally allocated to the selected kebeles based on the total number of eligible households found in each kebele. Systematic sampling method was used to identify participants’ households from each kebele. When there were two or more eligible children in the selected household, one child was selected by lottery method to be included in to the study (Fig. 1).

### Data collection

Quantitative data were collected using structured questionnaire. Interviewer administered was used to collect socio-demographic data of mother/caregiver, child characteristics and child caring/feeding practices. The questionnaire was prepared in English language after different literature review and translated into *Sidamigna* language for data collection. Three female clinical nurses and three Health Extension Workers (HEWs) were recruited to collect data and three public health professionals in nutrition were recruited to supervise the data collection process in addition to investigators. For ensuring data quality two days training was provided for data collectors and supervisors. Anthropometric data were collected through measurement of length/height and weight of all children.

Weight and length/height of the children were obtained by anthropometric measurements. For children less than two years weight was measured using hanging weight scale and length was measured in recumbent position using wooden UNICEF height board. Weight for children older than two years was measured using seca digital weight scale to the nearest 0.1 kg and height was measured in standing position to the nearest1cm using UNICEF wooden height board. Calibration for the weight and height instruments was done upon every case examination. All measurements were taken twice and the mean value was used for data analysis. The child’s age was collected from vaccination cards. In case, there were no vaccination cards or any age records, a local calendar system (including *Chembelala*, *Genna* (charismas), *Fasika* (Easter), *Meskel* (the finding of true cross), *Enkutatesh* (Ethiopian new year), Remadan, Arefa and other locally known festivals) were used to estimate the child’s age.

### Study variables

**Dependent Variable**: The dependent variable in this study was undernutrition indicated by wasting, stunting and underweight status in children 6–59 months of age.

**Independent variables**: Four categories of factors were assessed as independent variables:

- *Socio-demographic variables*: Age of child and mothers, child sex, family size, household monthly income, maternal education and occupation and marital status of the mother
- *Child characteristics*: height, weight, birth order and childhood illness.
- *Child feeding practices*: exclusive breast feeding, colostrums feeding, duration of breast feeding, frequency of complementary feeding, bottle feeding and child immunization
- *Maternal characteristics*: number of children ever born and ANC visits [10].

### Data analysis

The data were exported to SPSS version 16 after it was coded and entered in the computer using Epi Info Version 6.04 statistical packages. Descriptive statistics was used to summarize the data and the results were presented using frequency tables and percentages. For the anthropometric measurements data analysis was performed using Emergency Nutrition Assessment (ENA) for SMART 2007 software. ENA for SMART 2007 software was used to convert the anthropometric measures; weight, height/ length and age values into Z-scores of the indices; Height- for-Age(HAZ), Weight-for-Height(WHZ) and Weight-for- Age(WAZ) taking sex into consideration. The WHO classification was used to classify the nutritional status of the children. A low height-for-age, below -2SD of the reference population indicates stunting while below -3SD indicates severe stunting. A low weight-for-height, below -2SD of the reference population, indicates wasting, while below − 3SD indicates severe wasting. A child with a weight-for-age below -2SD of the reference population is underweight while a child below -3SD is severely underweight [11].

Both bivariate and multivariable logistic regression analysis were employed to determine predictors of undernutrition. All variables found to be associated to undernutrition in bivariate analysis using Crude Odds Ratio with 95%CI at significant level of ≤ 0.2 were taken to multivariable regression analysis in order to control confounders. Adjusted Odds Ratio with 95%CI was used to determine degree of association between explanatory variables and nutritional status of the respondents. Statistical association between dependent and independent variables was declared significant at *p*-value of ≤ 0.05.

### Data quality management

The questionnaire was pre-tested on 5% of sample size in non selected kebeles to check the applicability of the questionnaire and feedback was used to make some modifications to the questionnaire [2]. Members of field staff were selected according to their qualifications and work experience in the field of data collection. Field staffs were given training before starting data collection. During training the objective of the study and method of data collection were discussed. Furthermore, each question included in the questionnaire was discussed in detail. The collected data were checked for its completeness and consistence each day by supervisors and investigators. Incomplete questionnaires were completed by making second visits to the homes. Data were also cleaned and rechecked after double data entry was performed. Weighing scales were calibrated and the scales indicator was checked against zero reading after weighing every child.

### Ethical consideration

Ethical clearance was obtained from Ethical Review Board of the Institute of Public Health, University of Gondar. Official letter of cooperation was written to Hawassa city administration health office by SNNPRs regional health bureau ethical review committee. Subsequently, permission from Tulla Sub city health office was obtained and each administration of the kebele was informed through letter from the sub city. Written and signed informed consent was obtained from each respondent after the purpose of the study and assurance of confidentiality was explained. It was explained to the participants that participation was voluntary. Participants with diarrhea, respiratory tract infections and undernutrition were referred to nearby health facilities for management.

## Results

### Socio-demographic characteristics of children and parents

From the total 834 proposed study respondents, complete response was obtained from 811 respondents making the response rate 97.2%. The mean age of participant children was 30.77 (SD ± 14.09) months for both sexes. Four hundred thirty two (53.2%) participant children were male. Five hundred eighty six (72.3%) of the children were born to mothers between 20 and 34 years. Fifty (6.2%) and 175 (21.6%) of the children were born from teenage mothers and mothers over 35 years of age respectively. Majority, 781 (96.3%) of the children were grown by both parents and the average household family size was 5.73 (SD ± 2). Nearly all of the study participants were Sidama by ethnicity 794(97.9%). In total, 554 (68.3%) of the mothers were housewife and only 444 (54.8%) were educated. Regarding religion, 720 (88.8%) of the children are belong to Christian families (Table 1).

**Table 1:** Socio-demographic characteristics of the sampled children and their mothers (*n* = 811), rural kebeles of Hawassa town, Southern Ethiopia, 2012

Variables	Frequency (%)
Sex of child	
Male	432(53.3)
Female	379(46.7)
Age of child	
6–11	58(7.2)
12–23	195(24)
24–35	159(19.6)
36–47	214(26.4)
48–59	185(22.8)
Age of mother	
Less than 20 years	50(6.2)
20–34 years	586(72.3)
More than or equal to 35 yrs	175(21.6)
Family size	
Less than 4 people	271(33.4)
4–6 people	170(21)
More than 6 people	370(45.6)
Current marital status	
Single	30(3.7)
Married	781(96.3)
Religion	
Orthodox	2(0.2)
Protestant	694(85.4)
Catholic	24(3)
Muslim	91(11.2)
Ethnicity	
Sidama	794(97.9)
Others^a^	17(2.1)
Education of mother	
No formal education	367 (45.2)
Primary education	397(48.9)
Secondary & above	47(5.9)
Occupation of mother	
Farmer	141(17.4)
Housewife	554(68.3)
Merchant	71 (8.8)
Other^b^	45(5.4)

^a^Other- Oromo, Amhara, Gurage, Welaita and Hadiya

^b^Other- student, daily laborer, employee (Governmental and private)

### Healthcare utilization and child feeding practice

Five hundred ninety eight (73.7%) of the participated children were exclusively breastfed for the first six months whereas 213 (26.3%) given mixed feeding. Slightly more than half, 458(56.5%) of the children had ceased breastfeeding before two years of age. Cow’s milk, avocado juice and porridge were commonly used to start complementary feeding. Seven hundred thirty two (90.3%) of the children were fully immunized while 61 (7.5%) and 18(2.2%) children were partially immunized and not immunized at all respectively. Diarrheal disease is highly prevalent among the participant children as 715(88.2%) of them were reported to be experienced diarrhea within past 12 months. Among the total participated mothers, 292 (36%) were attended four ANC visit while 63 (7.8%) of mothers attended no ANC visit during most recent pregnancy. Six hundred sixty six (82.1%) of non primigravida mothers gave the next births after 24 months from the previous birth. Nearly two third of the households 528 (65.1%) in the study area had only one under-five child while the rest 277(34.9%) had two or more under five children (Table 2).

**Table 2:** Maternal and child healthcare utilization and child feeding practices (*n* = 811), Rural Kebeles of Hawassa, 2012

Variables	Frequency (%)
Breast feeding type (first six months)	
Exclusive	598(73.7)
Mixed	213(26.3)
Cessation of breast feeding	
Before two years	458(56.5)
Two years and beyond	353(43.5)
Feed on colostrum	
Yes	516(63.6)
No	295(36.4)
Frequency of daily complementary feeding	
At least 3 times per day	362(44.7)
Less than 3 times per day	449(55.3)
Immunization status	
Not immunized	18(2.2)
Partially immunized	61(7.5)
Fully immunized	732(90.3)
ANC visit	
At least one visit	748(92.2)
No visit at all	63(7.8)
Preceding birth interval	
Less than 24 months	145(17.9)
24 months and above	666(82.1)
Birth order of the last child	
First	128(15.8)
Second	143(17.6)
Third and more	540(66.6)
Number of under five children in HH	
One	528(65.1)
More than one	283(34.9)
Diarrheal morbidity in the past 12 months	
Yes	715(88.2)
No	96(11.8)

### Prevalence of undernutrition among children

Depending on the three anthropometric indices, height-for-age, weight-for- age and weight-for-height, the findings of this study revealed that 319(39.3%), 128(15.7%) and 51(6.2%) of the participant children were stunted, underweight and wasted respectively. In this study prevalence of severe stunting, severe underweight and severe wasting among the children were 98(12.1%), 34(4.2%) and 19(2.3%) respectively. In general, the prevalence of stunting (chronic undernutrition) increases as the age of a child increases, peaking at 36–47 months of age (11.1%). Female participants were more stunted than males. Underweight is also highest at the age group of 36–47 months (4.6%) and nearly equal male 65 (8.4%) and female 62(7.4%) were underweight. Slightly more males 29 (3.5%) were wasted than females 22(2.7%) (Table 3).

**Table 3:** Prevalence of severe and moderate malnutrition among six to fifty nine months of age (*n* = 811), rural kebeles of Hawassa town, 2012

**Nutritional Indices**	Frequency (%)							Total (%)
	Sex		Age in months					
	Male	Female	6–11	12–23	24–35	36–47	48–59	
Normal HAZ (HAZ ≥ -2SD)	198(24.4)	294(36.2)	38(4.7)	123(15.2)	98(12.1)	124(15.4)	109(13.4)	492(60.6)
Moderate stunting (-3SD ≤ HAZ < -2SD)	119(14.6)	102(12.7)	17(2.1)	56(6.9)	36(4.4)	63(7.7)	50(6.2)	221(27.3)
Severe stunting (HAZ < -3SD)	62(7.6)	36(4.5)	3(0.4)	16(1.9)	25(3.1)	27(3.4)	26(3.3)	98(12.1)
Normal WAZ (WAZ ≥ -2SD)	339(41.8)	344(42.4)	43(5.3)	174(21.5)	134(16.5)	177(21.8)	155(19.1)	683(84.2)
Moderate underweight (-3SD ≤ WAZ < -2SD)	49(6.1)	45(5.5)	12(1.5)	16(1.9)	18(2.2)	26(3.2)	22(2.8)	94(11.6)
Severe underweight (WAZ < -3SD)	19(2.3)	15(1.9)	3(0.4)	5(0.6)	7(0.9)	11(1.4)	8(0.9)	34(4.2)
Normal WHZ (WHZ ≥ -2SD)	370(45.6)	391(48.2)	54(6.7)	185(22.8)	145(17.9)	197(24.3)	179(22.1)	761(93.8)
Moderate Wasting (-3SD ≤ WHZ < -2SD)	18(2.2)	14(1.7)	4(0.5)	6(0.7)	9(1.1)	10(1.2)	3(0.4)	32(3.9)
Severe wasting (WHZ < -3SD)	11(1.3)	8(1.0)	0(0.0)	4(0.5)	5(0.5)	7(0.9)	3(0.4)	19(2.3)

### Predictors of undernutrition

Bivariate and multivariate logistic regression analysis was conducted to determine factors predicting undernutrition. Predictors of the three types of undernutrition, stunting, underweight and wasting were analyzed separately. The result indicated that female children [AOR = 0.40; 95%CI (0.21, 0.86)], feeding breast for at least two years [AOR = 0.48; 95%CI (0.12, 0.88)] and those who have not experienced diarrheal morbidity in the past 12 months [AOR = 0.42; 95%CI (0.15,0.94)] were less likely to be stunted as compared with their counterparts. However, children who fed complementary feeding for fewer than three times per day were 3 times [AOR = 3.01; 95%CI (1.48, 5.58)] more likely to be stunted than those who fed at least three times per day. Participant children who didn’t receive colostrum were 1.26 times [AOR = 1.26; 95%CI (1.09, 2.87)] more likely to be stunted than children who received colostrum (Table 4).

**Table 4:** Factors associated with stunting among children aged between six to fifty nine months of age (*n* = 811), rural kebeles of Hawassa town, 2012

Explanatory Variables	Stunting		COR 95% CI	AOR 95% CI	*P*-value
	Yes	No			
Sex of the child					
Male	114	318	1.00	1.00	
Female	205	174	0.31(0.12,0.83)	0.40(0.21, 0.86)^a^	<0.001
Age of mother					
35 years and less	274	362	1.00	1.00	
Older than 35 years	45	130	2.18(1.42,4.10)	1.75(1.15,3.86) ^a^	0.03
Colostrum feeding					
Yes	213	303	1.00	1.00	
No	106	189	1.25 (1.04, 3.18)	1.26 (1.09, 2.87)^a^	0.02
Exclusive BF in the first six months					
Yes	248	350	1.00	1.00	
No	71	142	1.42 (1.12, 5.25)	1.34 (1.23, 3.92)	0.18
Cessation of breast feeding					
Before two years	258	200	1.00	1.00	
Two years and beyond	234	119	0.65(0.10,0.92)	0.48(0.12,0.88)^a^	<0.001
Frequency of complementary feeding					
At least 3 times per day	198	164	1.00	1.00	
Less than 3 times per day	121	328	3.27(1.23, 6.41)	3.01 (1.48, 5.58)^a^	0.01
Diarrheal morbidity in the past 12 months					
Yes	274	441	1.00	1.00	
No	45	51	0.70(0.10,0.97)	0.42(0.15,0.94)^a^	<0.001

^a^Statistically significant association

Regarding underweight household family size being more than four were 4.16 times (AOR = 4.16; 95% CI (2.47, 9.68) more likely to be underweight than children from family size of less than four. Frequency of complementary feeding is significantly associated with undernutrition as children who fed less than three times per day were 2.87 times [AOR = 2.87; 95%CI (1.62, 5.38)] more likely to be underweight than those who fed at least three per day. Children who didn’t face diarrhea in the past 12monthsprior to the data collection were less likely to develop underweight than children with diarrheal disease [AOR 0.28; 95%CI (0.15, 0.74)]. Risk of underweight among children whose mothers have no formal education was 0.32 times less likely than children whose mothers have formal education [AOR 0.68; 95%CI (0.30, 0.93)] (Table 5).

**Table 5:** Factors associated with underweight among children aged between six to fifty nine months, rural kebeles of Hawassa town, 2012, (*n* = 811)

Explanatory Variables	Underweight		COR 95% CI	AOR 95% CI	*P*-value
	Yes	No			
Sex of the child					
Male	69	363	1.00	1.00	
Female	59	320	0.87(0.42,0.93)	0.84(0.21, 2.86)	0.08
Maternal formal education					
No formal education	49	318	1.00	1.00	
Have formal education	79	365	0.71 (0.28, 0.96)	0.68 (0.30, 0.93)^a^	<0.001
Family size					
Less than four	82	189	1.00	1.00	
Four and above	46	494	4.65(2.89,11.30)	4.16(2.47,9.68)^a^	0.02
Exclusive BF in the first six months					
Yes	57	541	1.00	1.00	
No	71	142	0.21 (0.02, 0.75)	0.38 (0.13, 2.72)^a^	0.03
Cessation of breast feeding					
Before two years	66	392	1.00	1.00	
Two years and beyond	62	291	0.79(0.28,0.92)	0.71(0.32,0.88)^a^	<0.001
Frequency of complementary feeding					
At least 3 times per day	87	275	1.00	1.00	
Less than 3 times per day	41	408	3.14(1.23, 5.41)	2.87 (1.62, 5.38)^a^	0.01
Practiced bottle feeding					
Yes	28	229	1.00	1.00	
No	100	454	0.55 (0.09, 0.82)	0.47 (0.14, 1.36)	0.07
Diarrheal morbidity in the past 12 months					
Yes	97	618	1.00	1.00	
No	31	65	0.32(0.10,0.84)	0.28(0.15,0.74)^a^	<0.001

^**a**^Statistically significant association

Regarding wasting, it was observed that the likelihood of being wasted was significantly higher for children who were born within 24 months of the preceding sibling. As compared with children who were born within 24 months from the preceding sibling, the risk of wasting was 1.21 times [AOR = 1.21 95%CI (1.11, 2.34)] more likely for children who were born after 24 months. Number of under five years children in the household was also found to be statistically associated with underweight evidenced with that children who were from households with more than one under five children were 1.48 times [AOR = 1.4; 95% CI (1.16,4.82)] more likely to be wasted compared to children from households with one under five children. Children who didn’t practice bottle feeding were less likely [AOR = 0.52; 95%CI (0.14, 0.86)] to be wasted compared with those who practiced bottle feeding (Table 6).

**Table 6:** Factors associated with wasting among children aged between six to fifty nine months, rural kebeles of Hawassa town, 2012, (*n* = 811)

Explanatory Variables	Wasting		COR 95% CI	AOR 95% CI	*P*-value
	Yes	No			
Preceding birth interval					
24 months and above	43	623	1.00	1.00	
Less than 24 months	8	137	1.18 (1.08, 2.56)	1.21 (1.11, 2.34)^a^	0.03
Number of under-five children in the HHs					
One child	38	490	1.00	1.00	
More than one children	13	270	1.61(1.21,6.85)	1.48(1.16,4.82)^a^	0.006
Colostrum feeding					
Yes	35	481	1.00	1.00	
No	16	279	1.26 (1.14, 3.28)	1.22 (1.10, 2.97)^a^	0.04
Frequency of complementary feeding					
At least 3 times per day	29	333	1.00	1.00	
Less than 3 times per day	22	427	1.69(1.19,5.11)	1.58(1.23,4.48)^a^	0.01
Cessation of breast feeding					
Before two years	22	436	1.00	1.00	
Two years and beyond	29	324	0.56(0.10,0.92)	0.48(0.12,0.88)^a^	<0.001
Practiced bottle feeding					
Yes	11	246	1.00	1.00	
No	40	514	0.57 (0.19, 0.92)	0.52 (0.14, 0.86)^a^	<0.001

^a^Statistically significant association

## Discussion

This study revealed high prevalence of child undernutrition in the study area. The finding showed that 39.3% of the children were stunted. Prevalence of underweight and wasting was found to be 15.7% and 6.2%) respectively. The finding of our study showed that child sex, diarrheal morbidity, mothers’ educational status, family size, frequency of daily complementary feeding, age at initiation of complementary feeding, colostrum feeding; preceding birth interval and bottle feeding practice were found to be significant predictors of undernutrition.

The finding of our study indicated lower prevalence of undernutrition (stunting, underweight and wasting) in comparison with the 2011 EDHS report in which 44%, 29% and 10% of the children were stunted underweight and wasted respectively [6]. This study also indicated lower prevalence of undernutrition among children than a number of studies conducted in different parts of the country [2, 8, 12–15]. This lower prevalence of undernutrition could be attributed to that the study area is green and different fruits and vegetables that are rich with different nutrients are harvested in the area. However, prevalence of undernutrition in our study was higher than a comparative cross sectional study conducted in Nepal (except for wasting) in which prevalence of underweight, stunting and wasting was reported as 27%, 37% and 11% respectively [16]. Our research finding also showed higher prevalence of undernutrition than other studies conducted in different parts of Ethiopia [10, 17–19]. As observed from these literatures, there were improvements of undernutrition over time. This could be attributed to the efforts of the health sector to enhance good nutritional practices through health education and provision of micronutrients to the most vulnerable group. In addition, the health extension programme has included nutrition as one part of its packages.

The finding of this study showed that the prevalence of undernutrition increases with age. Prevalence of all forms of undernutrition in the younger age group was lower as compared with older children peaking at the age groups of 36–47 months. Our finding is consistent with a study conducted in northern part of Ethiopia in which undernutrition was found to be peak (66.7%) at the age of 12–23 months [20]. It is also inconsistence with the study conducted at Butajira where undernutrition was reported to be peaking at 12 months (21.2% underweight, 48.1% stunted and 8.4% wasted) than 6 months (21.7% underweight, 26.7% stunted and 16.7% wasted) [21]. It is also consistent with the results of researches in other African countries Congo [22] and South Africa where underweight was associated with age less than 12 months [23]. This might be due to the protective effect of breast-feeding against malnutrition, since breast feeding is universal in Ethiopia as almost all (90%) children are breast-fed and most continue to breast-feed during their first year of life [6].

Parental socio demographic variables are found to be independently associated with children’s undernutrition. Maternal age was significantly associated with children’s undernutrition as children born to mothers aged 35 years and above were more likely to be stunted as compared to those who born to mothers aged less than 35 years. This finding is similar to the finding of study conducted in the Ethiopia [13]. Children of mothers with no formal education were more likely to be underweight when compared with children of educated mothers. This is consistent with study conducted in Ethiopia, [2, 24] Democratic Republic of Congo [22] and Bangladesh and Pakistan [23, 25] which showed parental education status was positively associated with child undernutrition. It is obvious that educated mothers are more autonomous to make decisions on resource allocation to nourish their children. It may also be attributed to better childcare practices adopted by educated mothers than those by uneducated mothers.

Birth interval was independently associated with wasting as children born within 24 months of the preceding siblings were more likely to be wasted than those who born after 24 months. This study also identified that children born to a household with more than four family size were more likely to be underweight when compared to children from a household with less than four family size. This finding is in line with the study conducted in Vietnam and Bangladesh [22, 25]. This could be because families with more children experience more economic strain for food consumption and hence they are more likely to suffer from poor nutritional status.

The children’s individual factors were also found to be independently associated with undernutrition among children. Sex of the child and diarrheal morbidity were found to be significantly associated with stunting. Presence of diarrheal morbidity in the last one year prior to data collection period was significantly associated with stunting and underweight. The results of this study are in agreement with the results of studies conducted in different developing countries [26–29]. This is because diarrhea may result in lower appetite and poor digestion and mal-absorption. Compared to boys, the likelihood of stunting was lower among girls. Similarly, many studies in Ethiopia and elsewhere have reported that under-five male children are more likely to become stunted than their female counterparts [8, 29–32]. This could be because of boys are more influenced by environmental factors including diet than girls [30]. Birth interval and bottle feeding practice are independently associated with wasting. Children who were born in less than 24 months of the preceding birth were more likely to be wasted as compared with children born beyond 24 months. Children who practiced bottle feeding were more likely to be wasted than those who didn’t practice bottle feeding. The results of this study are in agreement with results of studies conducted in Ethiopia [3, 8]. This could be attributed to that bottle feeding is one of the factors that can result in diarrhea which end in undernourishment.

Sub-optimal early child feeding practices were also found to be statistically associated with nutritional status of children. Children breastfed for less than 2 years were more likely to be undernourished; stunted, underweight and wasted. The odds of stunting were lower among children who started complementary feeding at 6 months or later when compared with children started complementary feeding before the age of 6 months. Children who fed complementary feeding for less than three times per day were more likely to be stunted, underweight and wasted than their counterparts. This finding was consistent with the finding from similar study in which frequency of daily complementary feeding led to child stunting [2, 24, 30, 33]. This might be due to inadequate intake of nutrients from complementary foods that increases the risk of undernutrition. The finding could also be attributed that complementary feeding could be contaminated that may lead to diarrheal disease which inturn causes undernutrition. This study showed that children who didn’t feed on colostrum were more likely to be stunted and wasted than those who received it. Similar findings are reported in India [34] This is probably because colostrum are full of nutrients and antibodies which provide protective effect to the children and prevent them from infections that may cause malnutrition.

This study was suffered from different limitations. An important limitation of this study is the cross sectional nature of the design that poses difficulty to examine causal relationship among variables. Secondly, much of the data were obtained via self report which can lead to a potential recall bias to the events happened in the past about child’s history of illness and patterns of breastfeeding. Finally, information on some important confounders such as parasitic infection, HIV status and maternal nutritional status during pregnancy were not collected which can affected nutritional status of the children.

## Conclusion

This study revealed significantly high prevalence of undernutrition (stunting, underweight and wasting) among children aged six to fifty nine months in the study area. Undernutrition was found to be significantly associated with age and educational status of the mothers, family size, birth interval, early child’s feeding, bottle feeding practice and diarrheal morbidity. Much more effort should be undertaken at all levels to improve information, education and communication on awareness creation regarding young child feeding practice as per the national infant and young child feeding practice guideline. Further research should be conducted to investigate specific nutrient deficiency in body serum by using laboratory methods. Health care providers need to deliver quality nutrition interventions and mothers should be advised about nutrition during pregnancy in order to reduce the consequence undernutrition.

## Abbreviations

ANC: antenatal care
AOR: adjusted odds ration
CI: confidence interval
COR: crude odds ration
EDHS: Ethiopian demographic and health survey
ENA: emergency nutrition assessment
HAZ: height for age Z-score
HEP: health extension program
HIV: human immunodeficiency virus
SD: standard deviation
SNNPR: Southern Nation and Nationalities and People Region
SPSS: statistical package for social science
UNICEF: United Nations international children’s emergency fund
WAZ: weight for Age Z-score
WHO: World health organization
WHZ: height for weight Z-score
